# Serum N-Glycosylation in Parkinson’s Disease: A Novel Approach for Potential Alterations

**DOI:** 10.3390/molecules24122220

**Published:** 2019-06-13

**Authors:** Csaba Váradi, Károly Nehéz, Olivér Hornyák, Béla Viskolcz, Jonathan Bones

**Affiliations:** 1 Faculty of Materials Science and Engineering, Institute of Chemistry, University of Miskolc, 3515 Miskolc, Hungary; bela.viskolcz@uni-miskolc.hu; 2 Faculty of Mechanical Engineering and Information Technology, University of Miskolc, 3515 Miskolc, Hungary; aitnehez@uni-miskolc.hu (K.N.); oliver.hornyak@uni-miskolc.hu (O.H.); 3 Characterisation and Comparability Laboratory, NIBRT – The National Institute for Bioprocessing Research and Training, Foster Avenue, Mount Merrion, Blackrock, Co., Dublin A94 X099, Ireland; jonathan.bones@nibrt.ie; 4 School of Chemical and Bioprocess Engineering, University College Dublin, Belfield, Dublin 4 D04 V1W8, Ireland

**Keywords:** glycosylation, Parkinson’s disease, capillary electrophoresis, label-free quantitation, support vector machine

## Abstract

In this study, we present the application of a novel capillary electrophoresis (CE) method in combination with label-free quantitation and support vector machine-based feature selection (support vector machine-estimated recursive feature elimination or SVM-RFE) to identify potential glycan alterations in Parkinson’s disease. Specific focus was placed on the use of neutral coated capillaries, by a dynamic capillary coating strategy, to ensure stable and repeatable separations without the need of non-mass spectrometry (MS) friendly additives within the separation electrolyte. The developed online dynamic coating strategy was applied to identify serum N-glycosylation by CE-MS/MS in combination with exoglycosidase sequencing. The annotated structures were quantified in 15 controls and 15 Parkinson’s disease patients by label-free quantitation. Lower sialylation and increased fucosylation were found in Parkinson’s disease patients on tri-antennary glycans with 2 and 3 terminal sialic acids. The set of potential glycan alterations was narrowed by a recursive feature elimination algorithm resulting in the efficient classification of male patients.

## 1. Introduction

Parkinson’s disease (PD) is a long-term neurodegenerative disorder affecting millions of people worldwide with no accurate diagnostic marker [1]. PD is mainly linked with decreased dopamine production in the substantia nigra affecting the motor system [2]. Early symptoms of PD include rigidity, impaired balance and shaking, although dementia, depression, and anxiety can also be formed over time [3]. Due to the lack of an appropriate molecular diagnostic test, identification of PD patients is challenging and is based on the presence or absence of certain clinical features such as bradykinesia and postural instability [4,5]. This highlights the need for an appropriate molecular test that can be used as an alternative and to prevent patients from undergoing time-consuming clinical evaluation periods and facilitate diagnosis of the disease. Glycosylation of human serum proteins is reportedly altered in many inflammatory [6] and malignant diseases [7] and in neurodegenerative disorders such as Alzheimer’s [8] and PD [9]. The process of protein glycosylation is driven by several highly specific enzymes; therefore, the monitoring of glycosylation changes can provide potential markers of the altered intracellular biochemical processes [10]. The most favourable techniques to analyse protein glycosylation are liquid chromatography (LC) and capillary electrophoresis (CE) with fluorescence (FLR) and/or mass spectrometric (MS) detection [11]. Current LC-FLR methods are usually easily transferable to LC-MS, whereas in CE, modified methods are required due to the need of the non-MS friendly surfactants in CE-LIF (laser-induced fluorescence) [12]. Eliminating these additives from the separation electrolyte can result in the appearance of electro-osmotic flow leading to reduced reproducibility and problematic integration. Surface modification of silica capillaries using a polyethylene oxide non-covalent coating is reportedly an efficient alternative strategy to reduce electro-osmotic flow with acceptable long-term stability [13,14], suggesting a potentially new direction for CE-MS glycomics. The accurate MS-based quantitation of glycans is still challenging due to their structural heterogeneity and complexity. One of the most powerful strategies to quantify MS data is label-free quantitation where the expression of an examined feature can be measured based on the spectrometric signal intensity allowing the comparison of unlimited number of MS runs [15]. The generated data can be analysed by various statistical tests, although specifying the order of relevance of significant alterations can be problematic when the number of significant features is high. Support vector machine-estimated recursive feature elimination (SVM-RFE) is a suitable method for the recognition of relevant features which can improve cluster classification [16]. This method recursively selects the important features at the basis of the classification and evaluates classification accuracy resulting in a cross-validation score. Features with no contribution to accurate classifications are dropped; therefore, only a set of the most significant alterations remains. SVM-RFE has been shown to be a powerful tool in the identification of potential alterations and thus the classification of healthy and disease groups [17]. In this study, a novel CE-MS method was developed for the quantitative analysis of the serum *N*-glycome using commercially sourced serum samples from control and PD patients. Following development, the human serum *N*-glycome was characterised by CE-MS/MS in combination with exoglycosidase sequencing. Using the developed dynamic coating approach, 15 controls and 15 PD patient samples were analysed in triplicate and all 90 runs were aligned, normalised, and quantified by Progenesis QI. The exported abundances were used to perform statistical analysis where gender-associated alterations were found in male patients. The significant alterations were further narrowed by SVM-RFE, resulting in a clear classification of male patients.

## 2. Results and Discussion

### 2.1. Development of a Dynamic Capillary Coating for Robust CE-MS

In this study, a dynamic capillary coating-based CE-MS method was developed for the analysis of human serum glycosylation. During the associated method development, a specific focus was placed on ensuring that the developed electrolyte for capillary zone-based separations was MS compatible. Optimised parameters included the use of uncoated silica capillaries, with an ammonium acetate-based separation buffer and a capillary rinsing regime to ensure stable and repeatable separations without the requirements for non-MS friendly additives. As a first step, 0.2% polyethylene oxide (PEO, M_v_ 300,000) was mixed overnight with our previously developed [18] background electrolyte (30 mM ammonium-acetate, pH 5) and filtered through a 0.2 µm membrane. The capillary conditioning started with a basic wash with 2% *v*/*v* aqueous ammonia for 1 min, followed by an acidic wash with 2% *v*/*v* acetic acid for 1 min, and subsequent coating with the previously prepared 0.2% PEO 30 mM pH 5 ammonium-acetate for 3 min. Finally, a conditioning step was performed by the background electrolyte wash for 3 min. During all the conditioning steps, the capillary flow was switched to waste while 950 mbar was applied to the inlet vial. After conditioning, 1 min background electrolyte flush and sample injection (100 mbar 10 sec, ≈25 nL) was performed, followed by the application of −30 kV to the capillary for 50 min. Integrating the base peak chromatograms, we found that after each conditioning cycle six experimental runs could be made with high migration time reproducibility, that is, an average migration time %RSD of 0.65 was calculated for 10 peaks of the maltodextrin ladder (Table S1, Supplementary Materials), while the dynamic coating was stable (Figure S1, Supplementary Materials). After six runs, unwanted peak diffusion could occur (Figure S2, Supplementary Materials) due to the partial loss of the dynamic coating which resulted in a deterioration of migration time reproducibility and unstable current. Therefore, a wash and conditioning cycle was included after every six experimental injects for all subsequent experiments providing high reproducibility. The main advantage of this conditioning method is that regular inexpensive silica capillaries can be used for the analysis in combination with dynamic coating, enabling to bypass the use of PVA (poly-vinyl alcohol)-coated capillaries.

### 2.2. N-Glycan Structure Identification

**Figure 1:**
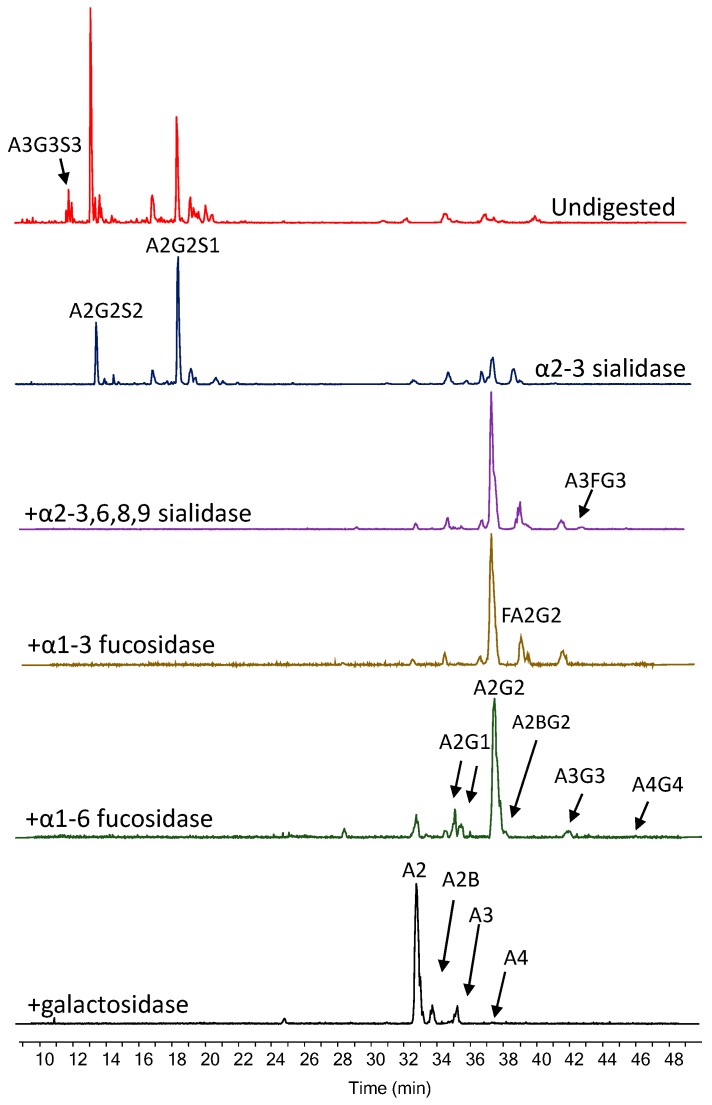
Exoglycosidase sequencing of 2-AA labelled human serum N-glycans by dynamic capillary coating-based capillary electrophoresis mass spectrometry.

The optimised dynamic capillary coating method was applied to characterise 2-AA labelled N-glycans using CE-MS/MS and subsequent exoglycosidase sequencing. Human serum glycans are mainly bi-, tri-, and tetra-antennary complex structures with different levels of sialylation and fucosylation. In order to identify these features, glycans were sequentially digested by α2-3 sialidase recombinant from *Streptococcus pneumonia*, α2-3,6,8,9 sialidase (*Arthrobacter ureafaciens*), α1-3/4 fucosidase (almond meal), α1-6 fucosidase (bovine kidney), α-galactosidase (coffee bean) and β-galactosidase (jack bean) (Figure 1). Data for each digest was collected in MS/MS mode providing essential fragmentation data of high abundant glycans (Figures S3–8, Supplementary Materials) for structural annotation. Glycan nomenclature was used as previously described [19]. As is shown in Figure 1, most of the A3G3S3 and A2G2S2 structures were found to be α2-3 linked, while A2G2S1 provided similar intensity compared to without digestion. After removing all sialic acids, core- (α1-6) and arm- (α1-3) fucosylated structures were also identified. Finally, the galactosidase digestion resulted in the main bi- (A2), tri- (A3) and tetra-antennary (A4) structures as expected, so no further digestion was needed. All digests were analysed by CE-MS/MS providing potential fragmentation patterns of the parent ions enabling the identification of the individual structures listed in Table 1.

### 2.3. Data Analysis

**Figure 2:**
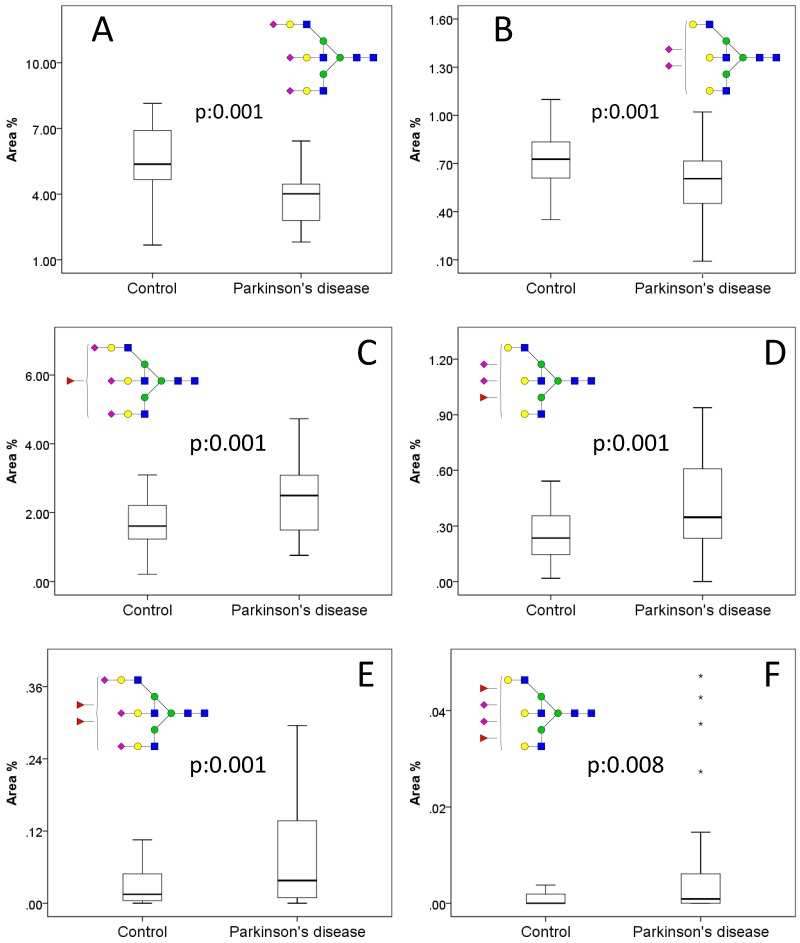
Significant serum N-glycan alterations in Parkinson’s disease on tri-antennary glycans with (**A**, **B**) no fucose, (**C**, **D**) 1 fucose, and (**E**, **F**) 2 fucoses.

The relative area percentages were calculated from the normalised abundances of the identified structures. As is shown in Table S2 (Supplementary Materials), 15 structures were significantly different when comparing the controls to the PD patients. One of the main trends between the two groups is the lower level of tri- (A3G3S3, A3G3S2, Figure 2A,B) and tetra-antennary glycans (A4G4S3, A4G4S2, Table S2, Supplementary Materials) with terminal sialic acids in PD. This result is in agreement with a recent study where lower sialylation was found on plasma IgG glycans in PD patients [9]. The lower level of A3G3S3 and A3G3S2 was also significantly different if one (Figure 2C,D) or 2 (Figure 2E,F) fucose residues were attached to the same structure, suggesting increased fucosylation in the disease. Similar alteration was reported in Alzheimer’s disease using lectin chip microarray where *Lotus tetragonolobus* lectin showed increased fucosylation in diseased patients [20]. To examine the origin of higher fucosylation in PD, control and disease groups were separated into control females, control males, PD females, and PD males resulting in the increase of significant features up to 26 using the Kruskal–Wallis test (Table S2, Supplementary Materials).

**Figure 3:**
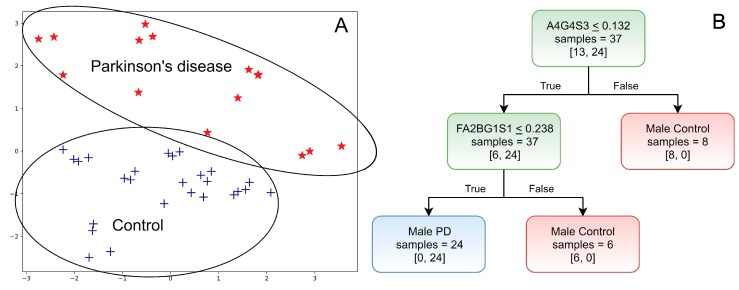
(**A**) Classification of male patients using recursive feature elimination and (**B**) the generated decision tree by support vector machine.

Due to the high number of significant alterations, SVM-RFE was applied to both female and male datasets. Analysing females, the highest cross-validation score was 0.63 when 11 features were selected by the algorithm (FA2, FA2BG1S1, FA2FG2S1, FA2G2S1, FA2G2S2, FA3G2S2, FA4G4S3, M4A1G1S1, M5A1G1, M5A1G1S1, M6) resulting in a poor classification as is shown in Figure S10 (Supplementary Materials). In males, 8 structures were selected with a cross-validation score of 0.86, suggesting a clear classification of control and PD groups (Figure 3A). Selected structures were as follows: A4G4S3, A4G4S3(2), FA2BG1S1, FA2G2, FA2G2S1, FA3G1S1, FA3G2S1, and FA3G3S1. As is shown in Figure 3B, the optimal decision tree was created by the evaluation of A4G4S3 and FA2BG1S1 showing efficient classification of control males and PD males. Similar to Figure 2, the sialylation and fucosylation were found to be the most important features. In the first level of the tree, 7 male controls were separated from 13 when the relative area percentage of A4G4S3 was higher than 0.132. The remaining group contained 6 control males and all the 24 PD males which were well distinguished by the evaluation of FA2BG1S1. When the relative area percentage of this structure was less than 0.238, the patient belonged to the PD group; if it was higher, it was a control patient. The main limitation of this evaluation is that the used structures are low abundant features and the precise quantitation of a structure with 0.1% relative area is challenging, although our main goal was to show the strength of this strategy in the classification of control and diseased patients.

## 3. Conclusions

A novel dynamic capillary coating strategy was presented in this study for CE-MS glycomics. The developed method was employed to annotate serum N-glycans in combination with exoglycosidase sequencing and MS/MS fragmentation. The identified features were quantified in patient samples by label-free quantitation. Altered levels of sialylation and fucosylation were found to be most significant in PD patients on tri- and tetra-antennary glycans, which were mainly originating from male patients. This alteration was also found to be critical in the classification of control and PD males. Our future target is to examine the efficacy of this classification strategy on a higher number sample set.

## 4. Materials and Methods

### 4.1. Chemicals and Reagents

Isopropanol, ammonium-hydroxide, acetic acid, sodium-cyanoborohydride, 2-aminobenzoic acid, dithiothreitol (DTT), iodoacetamide, and 10 kDa molecular weight cut-off (MWCO) centrifugal filters were purchased from Sigma-Aldrich (St. Louis, MO, USA). Exoglycosidase enzymes were obtained from Prozyme (San Francisco, CA, USA).

### 4.2. Patient Samples

For this study, 15 control (10 females and 5 males, average age 66.8) and 15 PD patient (6 females and 9 males, average age 66.5) serum samples were purchased from Bioreclamation IVT (Westbury, NY, USA).

### 4.3. N-Glycan Release, Labelling, and Clean-Up

Serum samples were normalised based on their protein concentration using Bradford protein assay. A 250 µg portion of serum samples from each patient was denatured using 8 M urea in 0.1 M tris buffer pH 8.0 (UA solution) and subsequently reduced and alkylated using 10 mM DTT and 55 mM IAA prepared in UA solution. The samples were buffer exchanged into 100 μL of 50 mM ammonium bicarbonate followed by N-glycan release using 500 units of PNGase F (New England Biolabs, Ipswich, MA, USA) overnight at 37 °C. The released glycans were collected from the deglycosylated proteins by centrifugation through 10 kDa MWCO filters and reduced to dryness using vacuum centrifugation (Thermo Fisher Scientific, Waltham, MA, USA). Glycans were reconstituted in 20 μL of 1% *v*/*v* aqueous formic acid to promote hydrolysis of the glycosylamines and reduced to dryness. A 5 µL portion of labelling solution (0.37 M 2-AA containing 1 M NaCNBH_3_ in DMSO/acetic acid (70:30)) was added to the dried sugars and incubated at 65 °C for 5 hours. HPLC water (10 µL) was added to the labelled samples and then purified using frontal HILIC (hydrophilic-interaction liquid chromatography) separation on a Thermo Scientific Ultimate 3000 UHPLC system (San Jose, CA, USA) and a 50.0 × 2.1 mm ID, 1.7 μm Waters BEH Glycan amide column (Milford, MA, USA). Samples were loaded onto the column and washed to remove excess 2-AA in 85% *v*/*v* acetonitrile. Purified glycans were eluted using 20% *v*/*v* aqueous acetonitrile, automatically collected and reduced to dryness using vacuum centrifugation prior to CE-MS analysis.

### 4.4. CE-MS Analysis

CE-MS experiments were made on an Agilent 7100 capillary electrophoresis system coupled to an Agilent G1607B orthogonal coaxial sheath sprayer and an Agilent 6520 Accurate-Mass Q-TOF mass spectrometer controlled by Mass Hunter B.07.00 (Santa Clara, CA, USA). Samples were injected by pressure (100 mbar, 10 seconds) and electrophoretic separations were performed by a constant voltage of −30 kV. Stable electrospray was supported by a sheath flow of 0.2% *v*/*v* ammonium hydroxide in 50% *v*/*v* water/isopropanol at 5 μL/min flow rate delivered by an Agilent G1376 capillary pump. During the analysis, 3.2 kV electrospray voltage was applied while the fragmentor voltage offset was 175 V. The drying gas temperature was 250 °C delivered at 5 L/min. Mass spectra were acquired using negative ionisation mode over the range of 500–2000 m/z with 2 GHz digitisation. MS/MS fragmentation spectra were recorded in data-dependent mode across the range of 100–2000 m/z, and 1 precursor ion was assorted for fragmentation with the absolute threshold of 200 [18].

### 4.5. MS Data Alignment and Analysis

Progenesis QI 2.1 (Nonlinear Dynamics, Newcastle, UK) was used for the alignment and normalisation of the 90 CE-MS runs where only doubly and triply charged ions were included. Sensitivity was set to absolute ion intensity and the minimum chromatographic peak width was 0.1 min. Mann–Whitney pairwise comparison and Kruskal–Wallis tests were performed by SPSS version 2.0 (IBM, Armonk, NY, US). Recursive feature elimination and decision trees were generated in Python 3.6, Scikit-learn-0.19.1 (Delaware, DE, US).

## Figures and Tables

**Table 1:** Annotated glycan structures by CE-MS/MS and exoglycosidase sequencing.

Digest	Structure	Charge State	Experimental Mass	Theoretical Mass	ppm
Serum_Undigested	A4G4S4	[M-3H]^3−^	1217.747	1217.7564	7.7
	A4FG4S4	[M-3H]^3−^	1266.4548	1266.4424	9.8
	FA4G4S4	[M-3H]^3−^	1266.4539	1266.4424	9.1
	FA4FG4S4	[M-3H]^3−^	1315.1101	1315.1283	13.8
	A3G3S3	[M-2H]^2−^	1499.0176	1499.0244	4.5
	A3FG3S3	[M-2H]^2−^	1572.0323	1572.0534	13.4
	FA3G3S3	[M-2H]^2−^	1572.0413	1572.0534	7.7
	FA3FG3S3	[M-2H]^2−^	1096.3801	1096.3858	5.2
	A4G4S3	[M-2H]^2−^	1120.7133	1120.7246	10.1
	A4FG4S3	[M-2H]^2−^	1169.4025	1169.4106	6.9
	FA4G4S3	[M-2H]^2−^	1169.4243	1169.4106	11.7
	A2G2S2(α2-3)	[M-2H]^2−^	1170.9056	1170.9106	4.3
	A2FG2S2	[M-2H]^2−^	1243.9346	1243.9396	4.0
	FA2BG2S2	[M-2H]^2−^	1345.47	1345.4792	6.8
	FA2G2S2	[M-2H]^2−^	1243.9321	1243.9396	6.0
	A3G3S2	[M-2H]^2−^	1353.4711	1353.4767	4.1
	A3FG3S2	[M-2H]^2−^	1426.5069	1426.5057	0.8
	FA3G3S2	[M-2H]^2−^	1426.4907	1426.5057	10.5
	FA3FG3S2	[M-2H]^3−^	1499.56	1499.5346	16.9
	A4G4S2	[M-2H]^2−^	1023.6952	1023.6928	2.3
	M3A1G1S1	[M-2H]^2−^	842.81	842.79	23.7
	M4A1G1S1	[M-2H]^2−^	923.844	923.823	22.5
	A4FG4S2	[M-2H]^2−^	1072.3606	1072.3787	16.9
	FA4G4S2	[M-2H]^2−^	1072.3604	1072.3787	17.1
	A2G2S1	[M-2H]^2−^	1025.3534	1025.3629	9.3
	A2FG2S1	[M-2H]^2−^	1098.3836	1098.3919	7.6
	FA2G2S1	[M-2H]^2−^	1098.3812	1098.3919	9.7
	FA2FG2S1	[M-2H]^2−^	1171.4225	1171.4208	1.5
	A3G1S1	[M-2H]^2−^	1045.90	1045.87	22.7
	A3G2S1	[M-2H]^2−^	1126.8932	1126.9026	8.3
	FA3G2S1	[M-2H]^2−^	1199.9208	1199.9315	8.9
	M5	[M-2H]^2−^	676.7294	676.7358	9.5
	M5A1	[M-2H]^2−^	778.29	778.2755	18.6
	M6	[M-2H]^2−^	757.7544	757.7622	10.3
	M7	[M-2H]^2−^	838.7827	838.7886	7.0
	M5A1G1	[M-2H]^2−^	859.3205	859.3019	21.6
	M8	[M-2H]^2−^	919.8172	919.8151	2.3
	M9	[M-2H]^2−^	1000.8345	1000.8415	7.0
Serum_NAN1	A2G2S2(α2-6)	[M-2H]^2−^	1170.9049	1170.9106	4.9
	A2G2S1(α2-6)	[M-2H]^2−^	1025.3599	1025.3629	2.9
Serum_NAN1_ABS	A3G3F	[M-2H]^2−^	1135.4051	1135.4102	4.5
	A4G4F	[M-2H]^2−^	1317.9564	1317.9763	15.1
Serum_NAN1_ABS_AMF	FA2	[M-2H]^2−^	790.7841	790.7913	9.1
	FA2G1	[M-2H]^2−^	871.8089	871.8177	10.1
	FA2BG1	[M-2H]^2−^	973.3743	973.3574	17.3
	FA2G2	[M-2H]^2−^	952.8348	952.8441	9.8
	FA2BG2	[M-2H]^2−^	1054.3739	1054.3838	9.4
Serum_NAN1_ABS_AMF_BKF	A2G1	[M-2H]^2−^	798.7831	798.7888	7.1
	A2G2	[M-2H]^2−^	879.8091	879.8152	6.9
	A3G3	[M-2H]^2−^	1062.3697	1062.3813	10.9
	A4G4	[M-2H]^2−^	1244.9375	1244.9474	8.0
Serum_NAN1_ABS_AMF_BKF_JBG	A1	[M-2H]^2−^	616.2136	616.2227	14.8
	A2	[M-2H]^2−^	717.7538	717.7624	12.0
	A3	[M-2H]^2−^	819.2926	819.3021	11.6
	A4	[M-2H]^2−^	920.8359	920.8417	6.3
Serum_NAN1_ABS_AMF_BKF_JBG_GUH	M3	[M-2H]^2−^	514.6796	514.683	6.6

## Supplementary Materials

The following are available online at https://www.mdpi.com/1420-3049/24/12/2220/s1, Figure S1: Reproducibility of 2-AA labeled maltodextrin ladder using dynamic mobile coating, Figure S2: Effect of stable coating on peak diffusion, Figure S3: MS/MS fragmentation of FA2, Figure S4: MS/MS fragmentation of FA2G2, Figure S5: MS/MS fragmentation of FA2G2S2, Figure S6: MS/MS fragmentation of A2, Figure S7: MS/MS fragmentation of A2G2, Figure S8: MS/MS fragmentation of A2G2S2, Figure S9: Increased fucosylation was originating from PD males, Figure S10: Female patients showed poor separation based on the selected features by RFE, Table S1: Calculated migration time reproducibility of 2-AA labeled maltodextrin ladder, Table S2: Significant differences between control and PD patients.
