# Supplementary material for: Serum N-Glycosylation in Parkinson’s Disease: A Novel Approach for Potential Alterations
Source: Molecules. 2019 Jun 13;24(12):2220. doi: 10.3390/molecules24122220 (PMC6630595; doi:10.3390/molecules24122220)

Supplementary Figure 1: Reproducibility of 2-AA labeled maltodextrin ladder using dynamic mobile coating

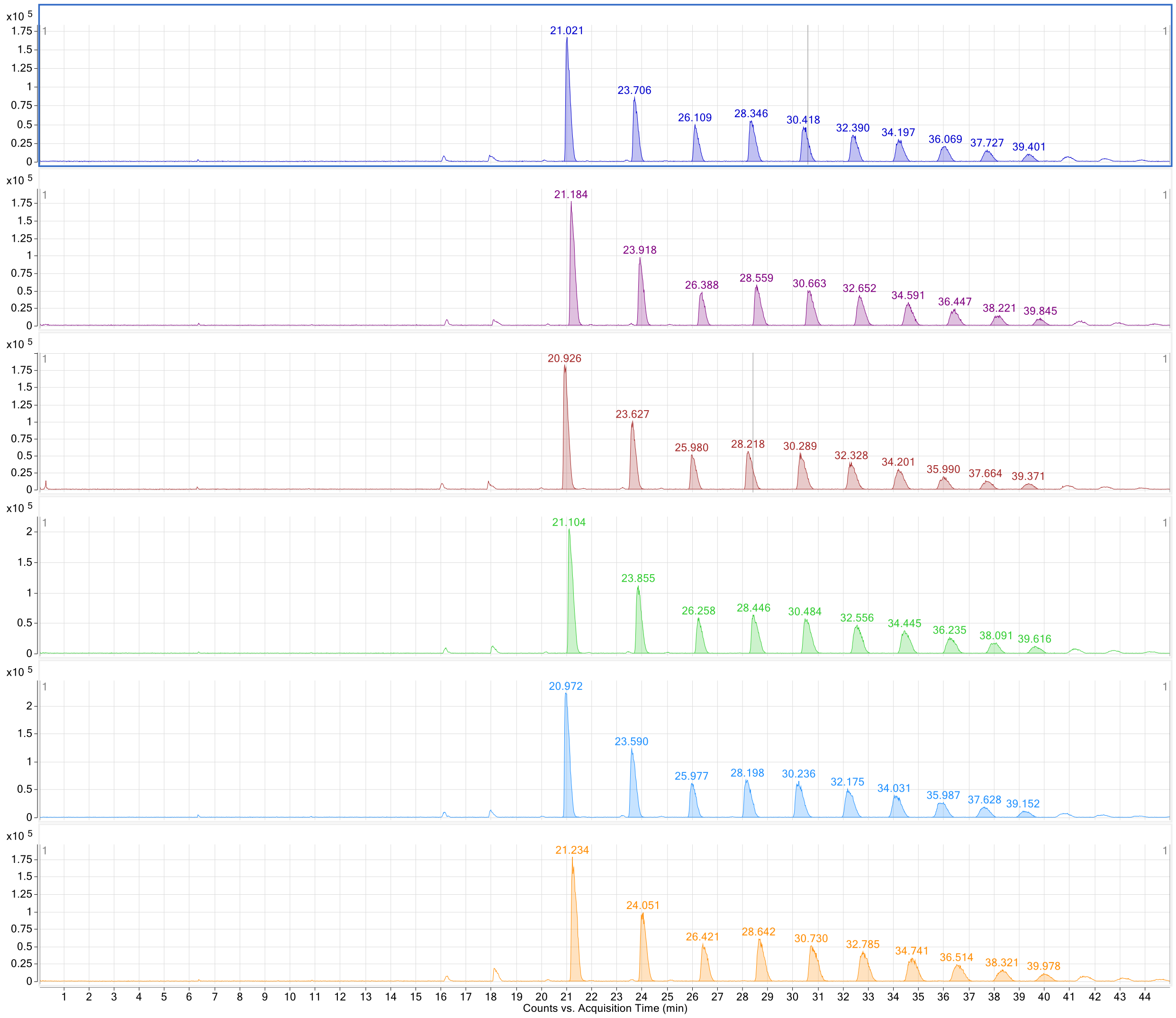

Supplementary Table 1: Calculated migration time reproducibility of 2-AA labeled maltodextrin ladder

	Ladder 1	Ladder 2	Ladder 3	Ladder 4	Ladder 5	Ladder 6	RT_AVG	RT_STDEV	RT_RSD%
**Peak 1**	21.02	21.18	20.93	21.10	20.97	21.23	21.07	0.11	0.53
**Peak 2**	23.71	23.92	23.63	23.86	23.59	24.05	23.79	0.16	0.69
**Peak 3**	26.11	26.39	25.98	26.26	25.98	26.42	26.19	0.18	0.69
**Peak 4**	28.35	28.56	28.22	28.45	28.20	28.64	28.40	0.16	0.58
**Peak 5**	30.42	30.66	30.29	30.48	30.24	30.73	30.47	0.18	0.59
**Peak 6**	32.39	32.65	32.33	32.56	32.18	32.79	32.48	0.21	0.63
**Peak 7**	34.20	34.59	34.20	34.45	34.03	34.74	34.37	0.25	0.72
**Peak 8**	36.07	36.45	35.99	36.24	35.99	36.51	36.21	0.21	0.58
**Peak 9**	37.73	38.22	37.66	38.09	37.63	38.32	37.94	0.28	0.73
**Peak 10**	39.40	39.85	39.37	39.62	39.15	39.98	39.56	0.28	0.72

**Average**									0.65

Supplementary Figure 2: Effect of stable coating on peak diffusion

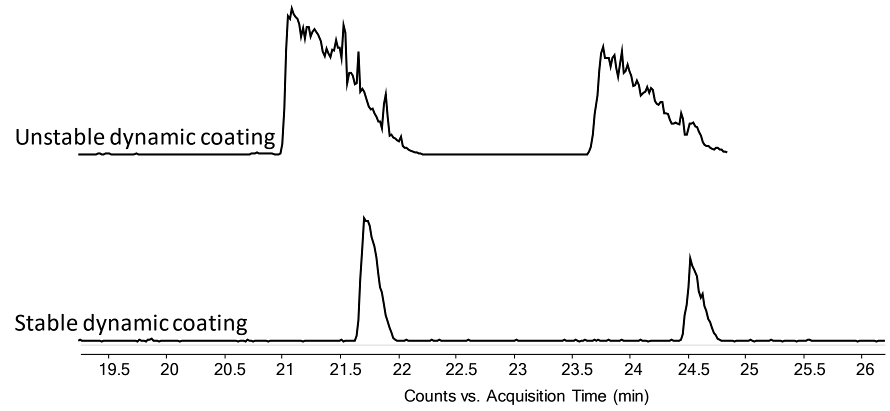

Supplementary Figure 3: MS/MS fragmentation of FA2

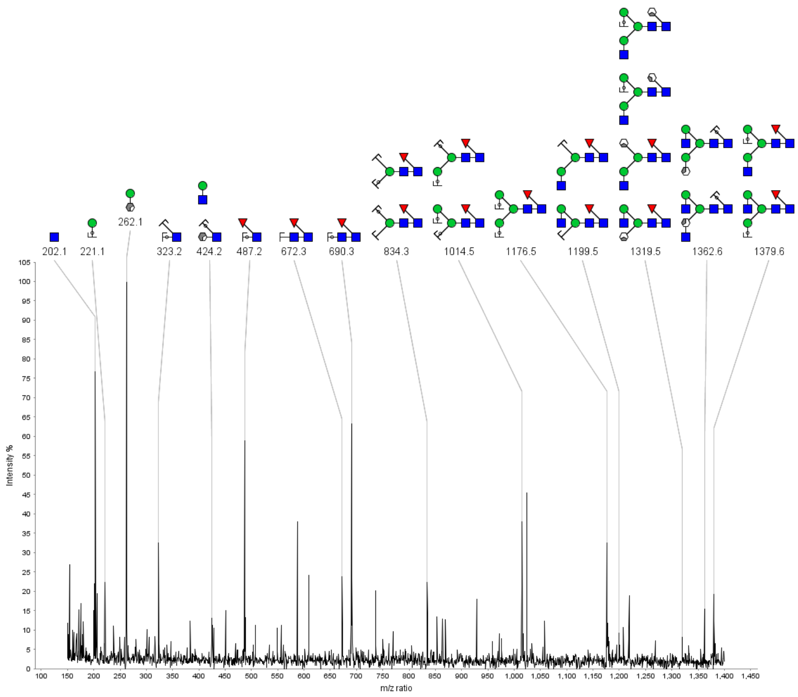

Supplementary Figure 4: MS/MS fragmentation of FA2G2

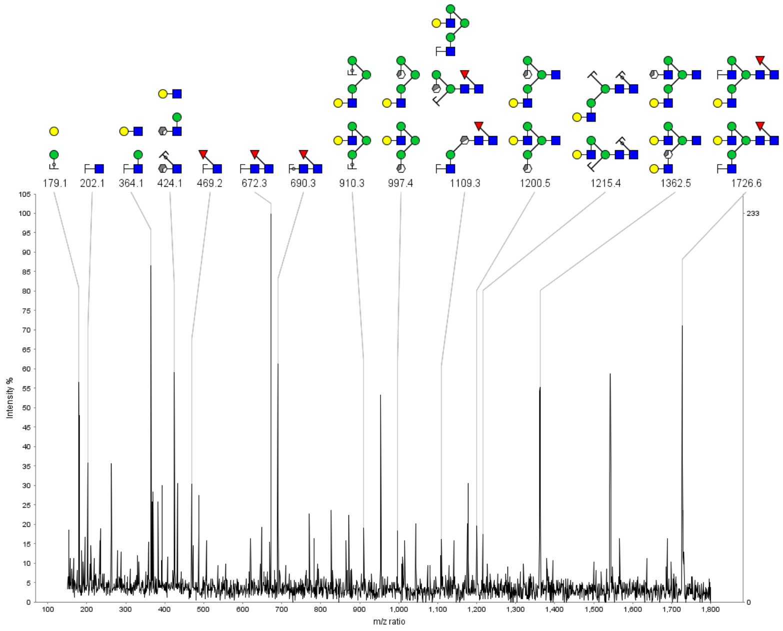

Supplementary Figure 5: MS/MS fragmentation of FA2G2S2

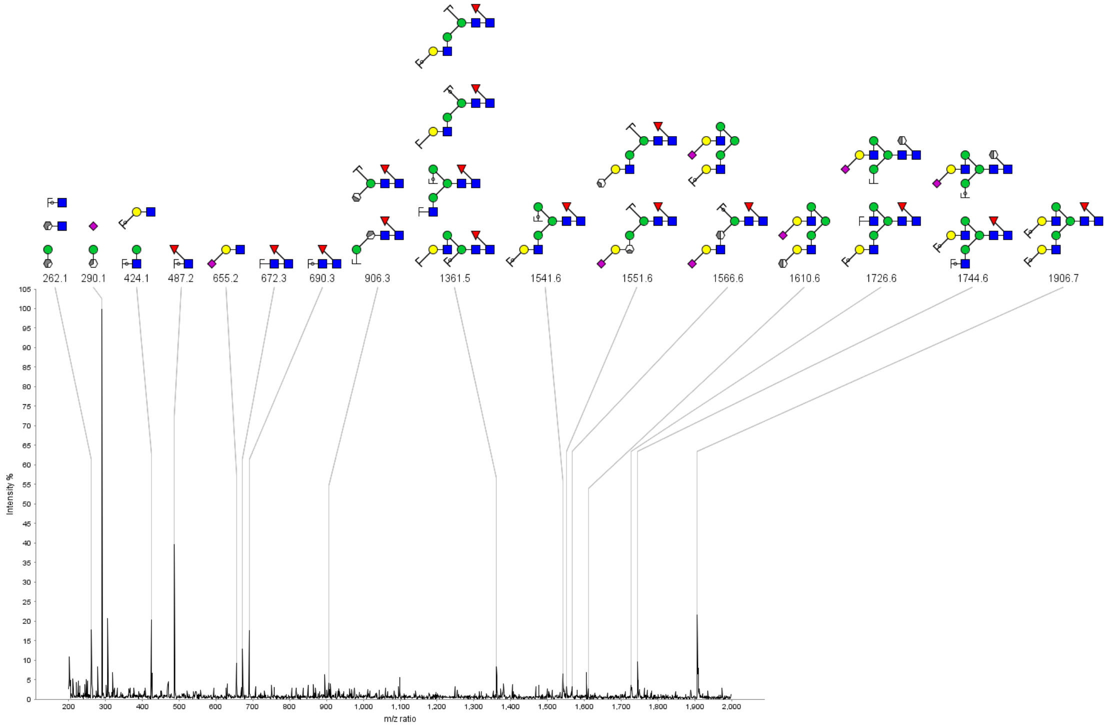

Supplementary Figure 6: MS/MS fragmentation of A2

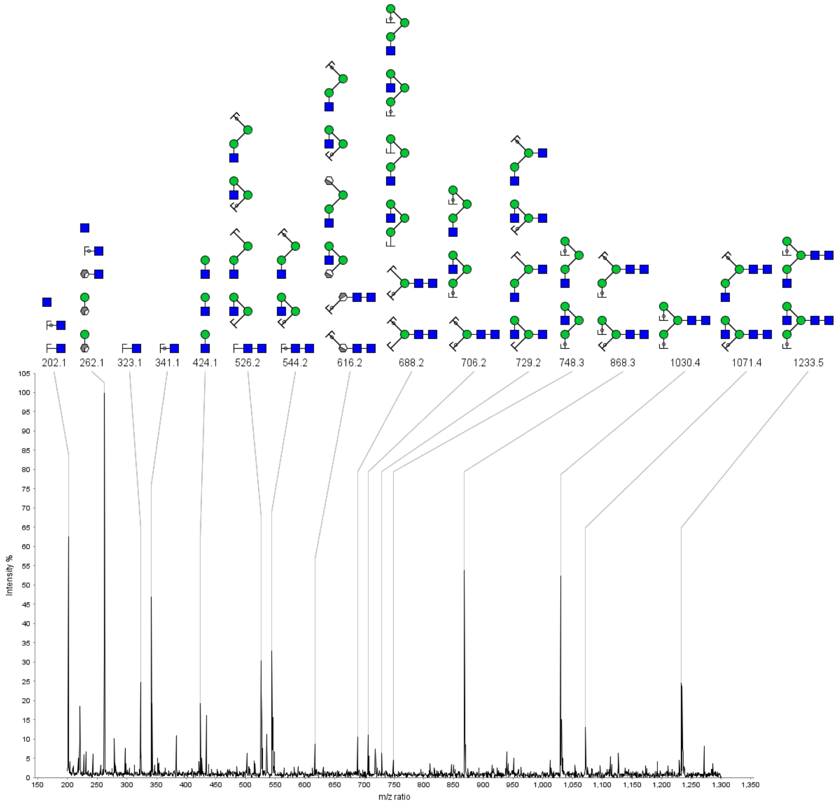

Supplementary Figure 7: MS/MS fragmentation of A2G2

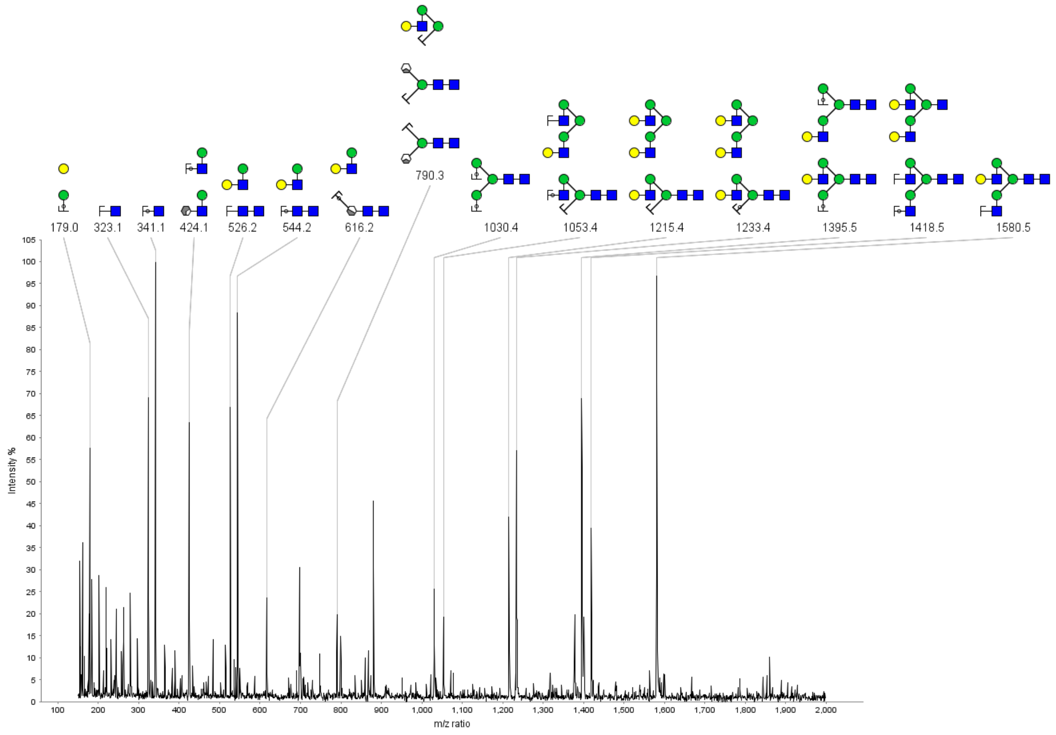

Supplementary Figure 8: MS/MS fragmentation of A2G2S2

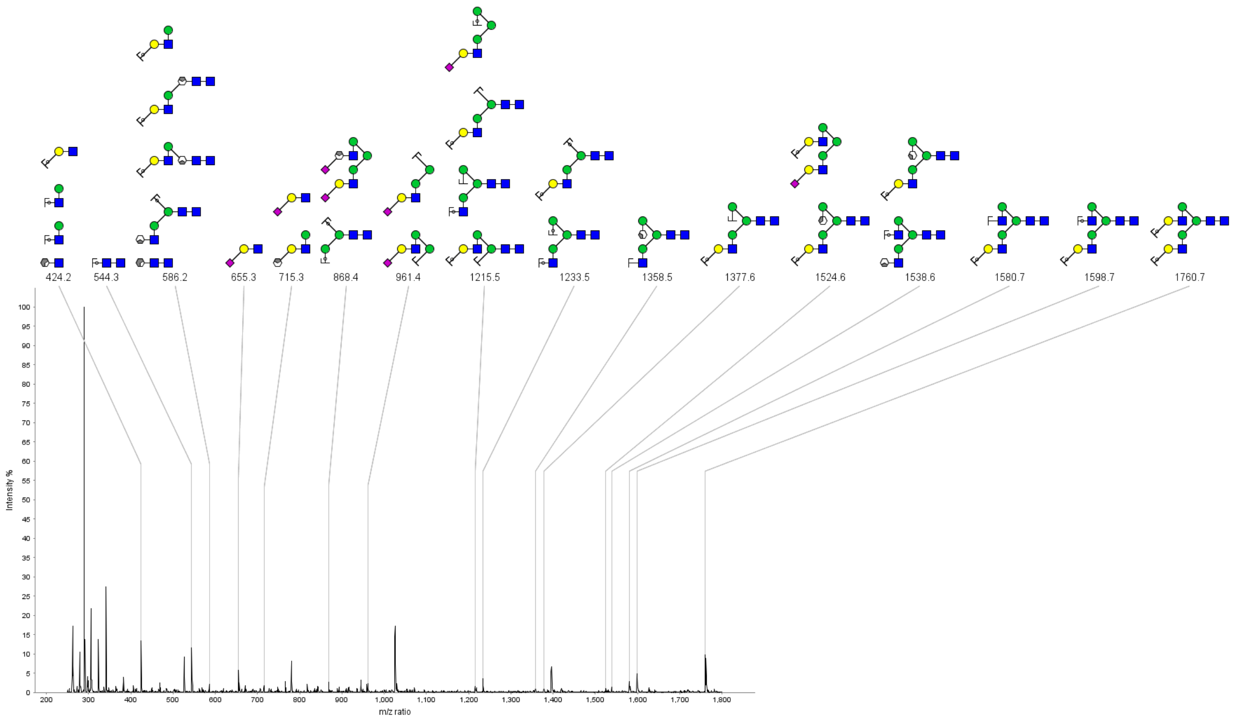

Supplementary table 2: Significant differences between control and PD patients

	Control		Parkinson's disease		Control vs PD	C_F vs C_M vs PD_F vs PD_M
	Mean	Std. Deviation	Mean	Std. Deviation	Significance level	Significance level
A4G4S4	0.11	0.10	0.10	0.08	0.12	0.01
A4FG4S4	0.02	0.03	0.02	0.04	0.01	0.01
A4G4S4_2	0.06	0.06	0.05	0.05	0.11	0.07
FA4FG4S4	0.00	0.00	0.00	0.01	0.00	0.00
FA4G4S4	0.01	0.03	0.02	0.02	0.02	0.08
FA4FG4S4_2	0.00	0.00	0.00	0.01	0.02	0.02
A3G3S3	5.53	1.66	4.80	1.77	0.00	0.00
A3FG3S3	1.70	0.74	2.10	1.03	0.00	0.00
FA3FG3S3	0.03	0.03	0.05	0.07	0.02	0.00
A4G4S3	0.14	0.08	0.11	0.08	0.00	0.00
A4G4S3_2	0.15	0.09	0.13	0.09	0.06	0.00
FA4G4S3	0.03	0.04	0.05	0.05	0.00	0.01
A2G2S2	44.29	6.75	44.88	6.50	0.50	0.31
A3G1S2	0.44	0.15	0.44	0.16	0.69	0.37
A3G2S2	0.05	0.06	0.05	0.05	0.78	0.00
FA2G2S2	4.97	1.43	4.96	1.24	0.98	0.16
FA3G2S2	2.02	1.09	1.96	0.86	0.76	0.17
FA2G2S2	0.24	0.15	0.25	0.13	0.34	0.02
FA2FG2S2	0.00	0.00	0.00	0.01	0.25	0.14
A3G3S2	0.75	0.23	0.66	0.27	0.00	0.00
A3G3S2	0.39	0.22	0.37	0.22	0.32	0.01
FA3G3S2	0.25	0.14	0.34	0.24	0.01	0.00
FA3FG3S2	0.00	0.00	0.00	0.01	0.01	0.01
A4G4S2	0.23	0.12	0.20	0.12	0.00	0.00
M3G1S1	0.31	0.09	0.31	0.11	0.70	0.74
M4G1S1	0.50	0.13	0.46	0.15	0.07	0.21
FA1G1S1	0.09	0.05	0.09	0.06	0.59	0.01
A3G1S1	0.36	0.27	0.34	0.22	0.38	0.42
M5G1S1	0.40	0.16	0.37	0.15	0.16	0.37
FA2G1S1	0.33	0.12	0.35	0.13	0.43	0.06
FA2BG1S1	0.09	0.08	0.08	0.07	0.88	0.04
A2G2S1	10.83	2.09	10.85	2.28	0.82	0.05
FA3G1S1	0.15	0.12	0.13	0.10	0.26	0.00
A3G1S1	0.06	0.05	0.06	0.04	0.84	0.74
A3G2S1	0.83	0.47	0.80	0.38	0.77	0.32
FA2G2S1	4.14	0.90	4.47	1.21	0.01	0.00
FA3G2S1	2.94	1.62	2.86	1.37	0.74	0.01
FA2FG2S1	0.04	0.03	0.04	0.03	0.12	0.30

A3G3S1	0.56	0.24	0.53	0.23	0.30	0.08
FA3G3S1	0.06	0.04	0.07	0.06	0.08	0.15
M5	1.45	0.64	1.44	0.68	0.68	0.07
A2	0.16	0.21	0.13	0.18	0.31	0.14
A2B	0.23	0.25	0.16	0.20	0.06	0.16
M5A1	0.04	0.05	0.05	0.06	0.36	0.04
M6	1.70	0.67	1.59	0.71	0.03	0.01
FA2	2.96	1.58	2.97	1.55	0.97	0.17
A2G1	0.23	0.25	0.21	0.24	0.56	0.69
FA2B	0.43	0.44	0.38	0.36	0.41	0.42
FA2BG1	0.21	0.22	0.17	0.17	0.39	0.34
M7	0.43	0.26	0.39	0.28	0.15	0.06
FA2G1	3.96	2.02	4.02	1.82	0.69	0.89
M5A1G1	0.09	0.08	0.08	0.07	0.69	0.52
FA2BG1	0.72	0.45	0.70	0.38	0.72	0.15
A2G2	0.39	0.33	0.42	0.37	0.60	0.90
A2BG2	0.06	0.07	0.06	0.06	0.75	0.54
M8	0.79	0.48	0.73	0.47	0.20	0.04
M9	0.93	0.63	0.83	0.55	0.35	0.02
FA2G2	1.72	1.40	1.87	1.34	0.13	0.26
FA2BG2	0.40	0.36	0.41	0.33	0.20	0.06

Supplementary Figure 9: Increased fucosylation was originating from PD males

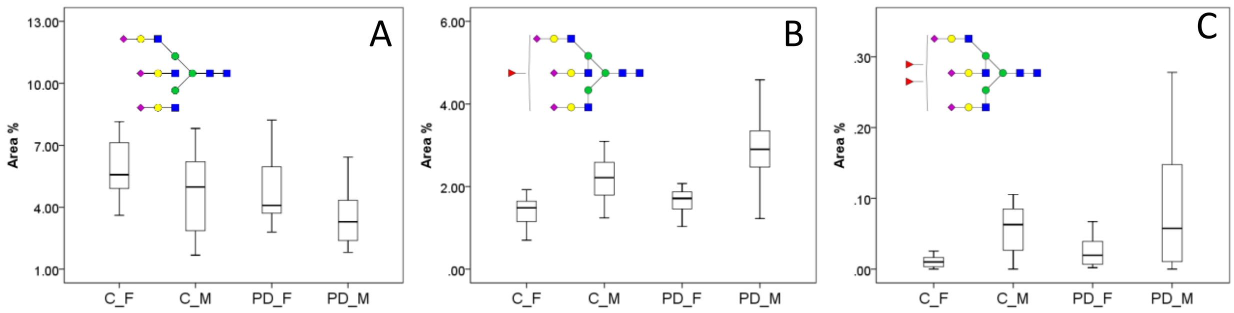

Supplementary Figure 10: Female patients showed poor separation based on the selected features by RFE

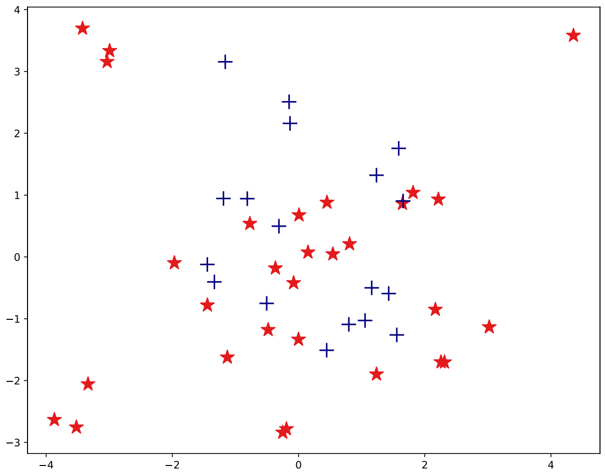

Supplement: Supplementary file 1 [file molecules-24-02220-s001.pdf]
